# Wildfires in Australia: a bibliometric analysis and a glimpse on ‘Black Summer’ (2019/2020) disaster

**DOI:** 10.1007/s11356-023-27423-1

**Published:** 2023-05-19

**Authors:** K M Shamsul Haque, Minhaz Uddin, Jeffrey Dankwa Ampah, Md. Kamrul Haque, Md. Shahadat Hossen, Md. Rokonuzzaman, Md. Yeamin Hossain, Md. Sazzad Hossain, Md. Zillur Rahman

**Affiliations:** 1 School of Agricultural, Environmental and Veterinary Sciences, Charles Sturt University, Wagga Wagga, NSW 2650 Australia; 2 Department of Environmental Science, Bangladesh Agricultural University, Mymensingh, 2202 Bangladesh; 3 State Key Laboratory of Engines, School of Mechanical Engineering, Tianjin University, Tianjin, 300072 China; 4 Institute of Bangabandhu War of Liberation Bangladesh Studies (IBLBS), National University, Dhaka, 1209 Bangladesh; 5 Department of Agricultural Extension Education, Sylhet Agricultural University, Sylhet, 3100 Bangladesh; 6 Department of Fisheries, Faculty of Agriculture, University of Rajshahi, Rajshahi-6205, Bangladesh; 7 Department of Agronomy and Haor Agriculture, Sylhet Agricultural University, Sylhet, 3100 Bangladesh; 8 Institute of Plant Nutrition and Soil Science, Kiel University, 24118 Kiel, Germany; 9 School of Life and Environmental Sciences, Sydney Institute of Agriculture, Faculty of Science, The University of Sydney, Sydney, Australia

**Keywords:** Australia, Wildfire, Bushfire, Bibliometric analysis, Biblioshiny

## Abstract

A wildfire, an unplanned fire that is mainly uncontrolled and originates in combustible vegetation in rural or urban settings, is one of the most pervasive natural catastrophes in some areas, such as Siberia, California and Australia. Many studies, such as standard reviews, have been undertaken to look into the works of literature on wildfires or forest fires and their effects on aquatic and terrestrial ecosystems. Regrettably, conventional literature reviews failed to identify the important researchers, evolving complexities, emerging research hotspots, trends and opportunities for further research on the ground of wildfire study. The present study employs bibliometric analysis to investigate this study area qualitatively and quantitatively. The Scopus database systems and Web of Science Core Collection yielded 78 qualifying papers, which were then evaluated using Biblioshiny (A bibliometrix tool of R-studio). According to the statistics, the discipline is expanding at a pace that is 13.68% faster than average. So far, three key periods of transformation have been documented: preliminary evolution (8 articles; 1999–2005), gentle evolution (14 articles; 2006–2013) and quick evolution (56 articles; 2014 to 2021). Forest Ecology and Management and Science journals have the highest number of publications, accounting for 7.70% of total wildfire-related articles published from 1999 to 2021. However, recent data indicate that investigators are shifting their focus to wildfires, with the term ‘Australia’ having the highest frequency (91) and ‘wildfire’ having the second highest (58) as the most appeared keywords. The present study will provide a foundation for future research on wildfire incidence and management by receiving information by synthesising previously published literature in Australia and around the world.

## Introduction

**Fig. 1:**
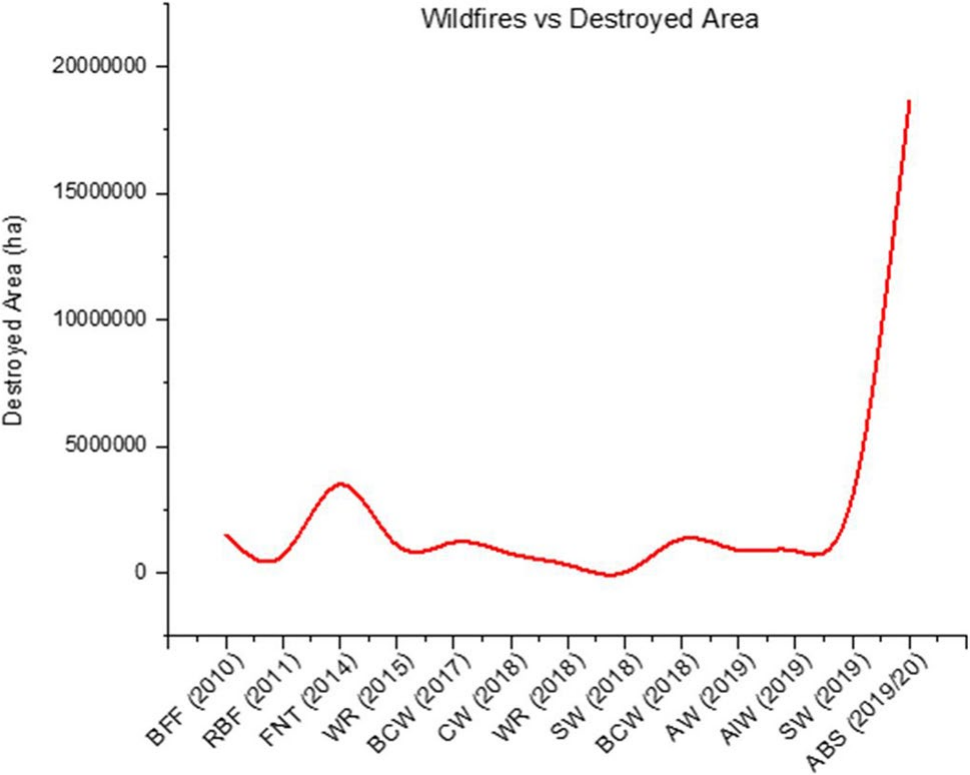
Global wildfires and damaged areas (Filkov et al. 2020a, b; Ward et al. 2020). Note: BFF, Bolivia Forest Fires; RBF, Richardson Backcountry Fire, Canada; FNT, Fires in Northwest Territories, Canada; WR, Wildfires of Russia; BCW, British Columbia Wildfires, Canada; CW, California Wildfires, USA; SW, Sweden Wildfires; BCW, British Columbia Wildfires, Canada; AW, Amazon Wildfires; AlW, Alberta Wildfires, Canada; SW, Siberian Wildfires, Russia; ABS, Australian Bushfire Season

Since its widespread distribution began 400–350 million years ago, fire has played a significant role in the dynamics of the global atmosphere and the evolution of biomes (Roach 2020; Haque et al. 2021). In fire-prone ecosystems, fire in the landscape (commonly termed wildfire, wildland fire or bushfire) has been considered as a ‘disaster’ when it engulfs the environmental components at a larger scale beyond control. Wildfires are a worldwide phenomenon that plays an important role in the terrestrial and atmospheric environments (Bowman et al. 2009). It has been around since the beginning of time, and rhyniophyte plant fossils that were preserved as charcoal caused the first known wildfire around 420 million years ago, during the Silurian epoch (Glasspool et al. 2004). Yearly, around 30–46 million km^2^ (approximately 4% of the total land surface) is burned (Randerson et al. 2012). Longer fire seasons are caused by changes in the environmental situation, which influence the frequency and intensity of wildfires (Westerling et al. 2006; Flannigan et al. 2013; Settele et al. 2015) and the wider area covered (Gillett et al. 2004). It all starts with a little site, which might have been caused by a lightning strike or human neglect. It spreads over a vast area of forested areas and locality and has adverse impacts on the environment, ecology, properties and human health. The abiotic and biotic constituents of the forest ecosystem are destroyed by wildfire (Godfree et al. 2021). At present, climate change and other associated factors are influencing more frequent and intense fires worldwide on a larger scale (Ward et al. 2020). Catastrophic fires have erupted in Australia, the USA, Brazil, and Russia in recent years, damaging on a larger scale (Fig. 1).

Like the USA, Russia, Brazil, Turkey, Italy and Canada, wildfire is typical in Australia almost yearly (Table 1). In Australia, a number of wildfire occurrences have been recorded, i.e. Gippsland fires and Black Sunday in 1926, Black Friday in 1939, the Australian Bushfire Season from 1974 to 1975, the Waterfall bushfire in 1980, recent Canberra bushfires in 2003 and the Black Saturday wildfire in 2009 are some examples of devastating wildfires that have occurred in recent history (Weber et al. 2019). The 2019–2020 ‘Black Summer’ wildfires were exceptional among others in terms of burned area, fatalities and ecosystem damages (Simmons et al. 2022; Wang and Cai 2020). This mega fire was 50 times more damaging than the historical worst wildfires in California and 5 times more extensive than the Amazon wildfires in 2019 (Ward et al. 2020). More than 15,000 fires occurred across all states of Australia, resulting in a catastrophe for aquatic and terrestrial ecosystems (Filkov et al. 2020a, b). This mega-fire destroyed a large number of flora, fauna and human habitats (Roach 2020). It killed 429 people due to smoke (Johnston et al. 2021), burned over 18 million hectares and damaged 3113 dwellings (Filkov et al. 2020a, b) and destroyed 3 billion animals (Van Eeden et al. 2020). Table 2 shows the overall impacts of Australia’s ‘Black Summer’ bushfire on air, water, soil, biodiversity, food and human health. The ‘Black Summer’ culminated in December–January, with significant wildfires consuming around double the total land area of preceding fire seasons throughout numerous states (Morgan et al. 2010), with 2019 being Australia’s warmest and driest year on history (Bureau of Meteorology (BoM) 2019). As a result, the cost of Black Summer crossed $110 billion, topping the $4.4 billion cost of the 2009 Black Saturday wildfires, leading to Australia’s maximum number of wildfire fatalities (Ell 2020). Large parts of eastern Australia were engulfed in smoke as a result of the Black Summer fires. A quarter of participants in a January 2020 survey in the worst-affected state of New South Wales (NSW) said wildfire smoke had harmed their health (The Australian Institute 2020). Emissions of fine particulate matter have been connected to negative health effects due to wildfire (Cascio 2018), with fatality rates rising on fire days with bad air quality (Morgan et al. 2010; Johnston et al. 2011). Table 3 shows the damages and fatalities caused by ‘Black Summer’ across Australia.

**Table 1:** Wildfire occurrences in different Australian states

**Year**	Wildfires	States	Burned area (ha)	Fatalities	Destroyed homes	References
1994	Eastern seaboard fires	New South Wales	400,000	4	225	BoM (2022)
1997	Dandenong bushfire	Victoria	400	3	41	CNN (1997)
1997	Perth and South-West Region bushfires	Western Australia	23,000	2	1	CNN (1997)
2002	Black Christmas bushfires	New South Wales	753,314	0	121	NSW Government (2007)
2003	Canberra bushfires	Australian Capital Territory	160,000	4	500	BoM (2022)
2003	Eastern Victorian alpine bushfires	Victoria	1,300,000	0	41	AIDR (2003)
2005	Eyre Peninsula bushfire	South Australia	77,964	9	93	Manton (2012)
2006	Jail Break Inn Fire	New South Wales	30,000	0	7	SMH (2006)
2006	Victorian bushfires	Victoria	160,000	4	57	The Courier (2007)
2007	2006–2007 Australian bushfire season	New South Wales, Victoria, Tasmania	1,360,000	5	83	Kennedy et al. (2006); Morton et al. (2006)
2007	Kangaroo Island bushfires	South Australia	95,000	1	0	Peace and Mills (2012)
2009	Black Saturday bushfires	Victoria	450,000	173	2029	CFA (2019)
2013	Warrumbungle bushfire	New South Wales	54,000	0	53	Van de Wetering (2013)
2013	New South Wales bushfires	New South Wales	100,000	1	208	RFS (2022)
2015	Esperance bushfires	Western Australia	200,000	4	10	Beattie and Baker (2015)
2015	Pinery bushfire	South Australia	85,000	2	91	AIDR (2016)
2016	Waroona Fire	Western Australia	69,165	2	181	BoM (2022)
2017	New South Wales bushfires	New South Wales	52,000	0	41	SBS News (2017)
2019	Tingha bushfire	New South Wales	23,419	0	19	BoM (2022)
2020	Black Summer bushfires	Nationwide	18,636,079	34	3051	Brulliard and Fears (2020); Noble (2020)

**Table 2:** Bushfire impacts after 2019–20 season in Australia

Affected components	Impacts
Air	○ Air quality has been destroyed in fire-affected states (Glover and Jessup 1999; Sastry 2002; Sapkota et al. 2005), and the Air Quality Index (AQI) has been escalated (Zhou 2019) ○ A NASA survey conducted in mid-December 2019 confirmed that the New South Wales and Queensland wild fires produced 250 million tonnes of CO_2_ since 1 August. NASA later reported that 306 million tonnes of CO_2_ had been released as of 2 January 2020 (Readfearn 2019) ○ The fine particles in the air in Sydney recognized worldwide as PM_**2.5**_ was measured at 734 μg (0.01133 g), which is the equivalent of 37 cigarettes (BBC News 2020), and smoke created a brown tint to the snow and the sky in Auckland turned orange (Haque et al. 2021) ○ In NSW, 9 weeks staring 1 November 2019, there were 247 early-stage foetus fatalities and 437 and 1535 hospitalizations for cardiovascular and respiratory diseases, respectively (Nguyen et al. 2021)
Soil	○ Bushfires destroyed millions of hectares of Australia’s land by damaging both above-ground vegetation and below-ground root masses as well as soil (AAS 2020) ○ The stored carbon in below-ground soils and vegetation (terrestrial carbon sinks) has been disturbed due to damage to above-ground vegetation (AAS 2020) ○ The 2019/2020 bush fire was strong enough to radiate heat to the underlying soil layers that disintegrated soil aggregates and soil organic matter; many essential nutrients and soil microorganisms were lost from the soil (Maunder 2019; AAS 2020)
Water	○ Cyanobacterial blooms and subsequent imbalanced aquatic ecosystem observed across Australia (DAWE 2021) ○ Organic matter, salts, and trace metals from ash of burned vegetation into water bodies reduced dissolved oxygen resulting in fish killing that was observed in Australia after 2019/2020 bushfire season (AAS 2020) ○ A downpour following the 2019 bushfires; significant amount of ash was introduced into surface water in NSW resulting in increased chlorine level (Ward et al. 2020)
Biodiversity	○ Around 143 million mammals, 2.46 billion reptiles, 181 million birds, and 51 million frogs were affected and at least 3 billion terrestrial vertebrates were displaced or destroyed (Van Eeden et al. 2020) ○ About 33% of Kangaroo Island Forest area was damaged which is the last habitat of Kangaroo Island dunnarts and Kangaroo Island glossy black cockatoos (DEW 2018; Brulliard and Fears 2020) ○ NASA reported that the number of dead koalas on the Kangaroo Island might be 25,000 or about 50% of the species’ total population (Dvorsky 2020) ○ One-quarter of Ligurian honey bee hives were ravaged that lived on Kangaroo Island (Khalil 2020) ○ After the megafirms, the Kangaroo Island assassin spider and the Kangaroo Island micro-trapdoor spider have not been found till the study findings were circulated, and it was believed that they might be fully destroyed or displaced (Marsh 2020; Haque et al. 2021)
Food safety	○ Agricultural production and average farm incomes have been recorded to have dropped by 8% in 2019/2020, about 4% below the 10-year average across Australia (ABARES 2020) ○ Australia’s total agricultural exports are projected to drop by 11% to 43 billion USD in 2019–2020 (Dvorsky 2020)
Human health	○ The most frequent complaints after the Black Summer bushfire season were eye and throat pain, coughing, and headaches (Borchers Arriagada et al. 2019) ○ According to a recent study, the bushfires smoke caused 2027 people to be admitted to hospitals with respiratory issues and 1305 people with asthma-related conditions (Borchers Arriagada et al. 2019) ○ Approximately 1100 people were hospitalized with cardiovascular complications caused by the fires (Duckett et al. 2020) ○ People suffered more than twice from post-traumatic stress disorder (PTSD), depression, and mental anxiety in ‘highly impacted’ communities where people died or where properties were damaged (Duckett et al. 2020)

**Table 3:** Fire and related losses of ‘Black Summer’ fires erupted in different states of Australia (Filkov et al. 2020a, b; Noble 2020; Wuth 2020)

State	Number of fires	Fatalities	Homes lost	Burned area (ha)
Victoria	3500	5	396	1,500,000
New South Wales	10,520	26	2448	5,500,000
Queensland	N/A	0	48	2,500,000
Tasmania	N/A	0	2	36,000
Western Australia	N/A	0	1	2,200,000
South Australia	1324	3	151	490,000
Northern Territory	N/A	0	5	6,800,000
Australian Capital Territory	N/A	0	0	86,464

*N/A* no data available

Australia is a habitat to 620,000 species, contributing to 7–10% of all species on the planet (Box 2020). Most of Australia’s species and ecosystems are found nowhere else on the planet. The Black Summer fires were termed by the Royal Commission into National Natural Disaster Arrangements (RCNNDA) as an ‘ecological calamity’, with ‘the most catastrophic habitat destruction for vulnerable species and damage of ecosystems in the postcolonial period’ (Wintle et al. 2020). More than 330 biological communities that were severely endangered and 37 biological communities that were threatened were destroyed by the fires; these communities are all protected under national environmental legislation. (RCNNDA 2020; Box 2020). The 2019/2020 bushfires also caused significant damage to vital ecosystems, such as clean water supplies. After a fire, the loss of plants and grasses, in addition to changes in the physicochemical properties of the soil, may greatly increase both the amount of surface runoff and the soil’s erodibility (Robichaud 2000; Shakesby and Doerr 2006; Shakesby 2011). Following rainfall, soil that has been eroded and ash pose a significant risk of contamination to aquatic systems and aquifers (Smith et al. 2011). Algal blooms can be aided by ash and degraded soil nutrients that release toxins that may induce carcinogenic and non-carcinogenic substances (Hohner et al. 2019).

### Background of the horror ‘Black Summer’ development

**Fig. 2:**
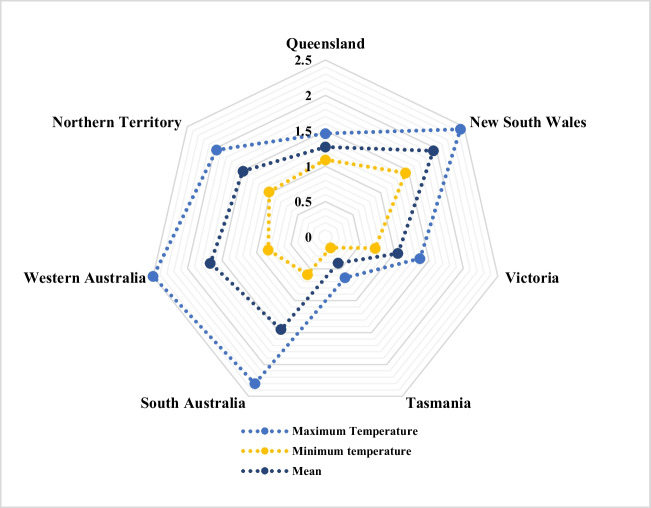
Temperature (°C) upsurge in Australian states in 2019–2020 bushfire season (ACS 2020)

According to the 2019 annual climate statement, the 2019/2020 Australian wildfire season was the hottest in recorded history, with a maximum temperature of + 2.09 °C and an average temperature of + 1.52 °C. It surpassed the previous average and maximum temperature records of + 1.33 °C and + 1.59 °C, respectively, in 2013. The mean minimum temperature change in Australia’s 2019/20 bushfire season was 0.95 °C, the sixth-warmest recorded value. Figure 2 shows variations in maximum and minimum temperatures at two key locations in NSW and VIC.

**Fig. 3:**
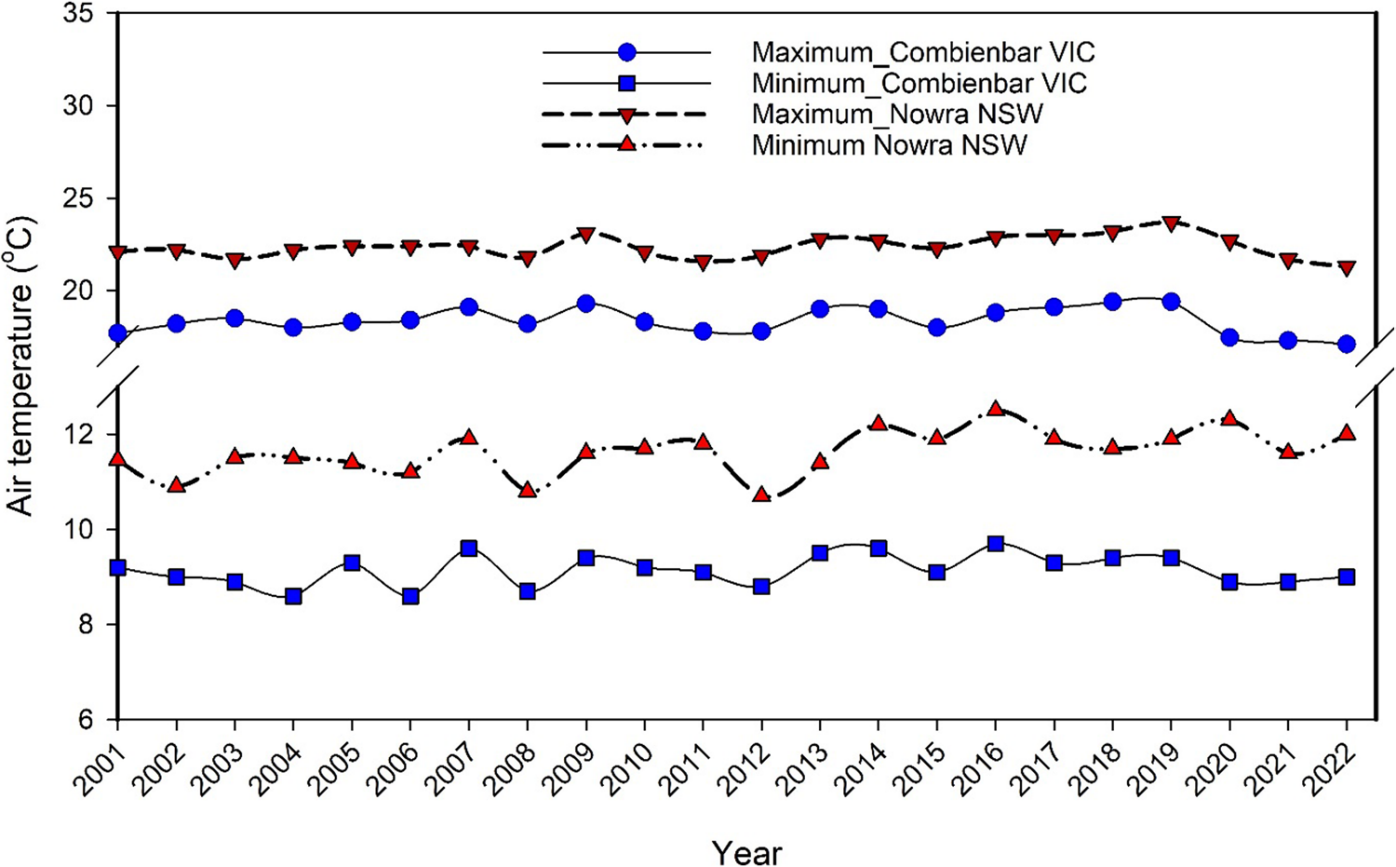
Mean maximum and minimum temperatures (°C) during 2001–2022 at Combienbar VIC and Nowra NSW. Data obtained from Australian Bureau of Meteorology

In addition, between 1999 and 2020, the average temperature in Australia exhibited a large range of variance. The temperature variation ranged from − 1.52 to 1.52°C above normal during the whole time (Fig. 3). The year 2000 had an average temperature of − 0.04°C, while the year 2019 saw an average temperature of + 1.52°C. The lowest average temperature ever recorded occurred in the year 2000 when it fell to − 0.04°C. Before the year 2005, the temperature was never higher than + 1°C. However, in 2005, it became the first year when it exceeded + 1°C, and after 2012 the mean temperature was higher than 1°C till 2020, except in 2015 and 2016 (ACS 2020). These high temperatures in Australia may have a favourable impact on the occurrence of bushfires.

**Fig. 4:**
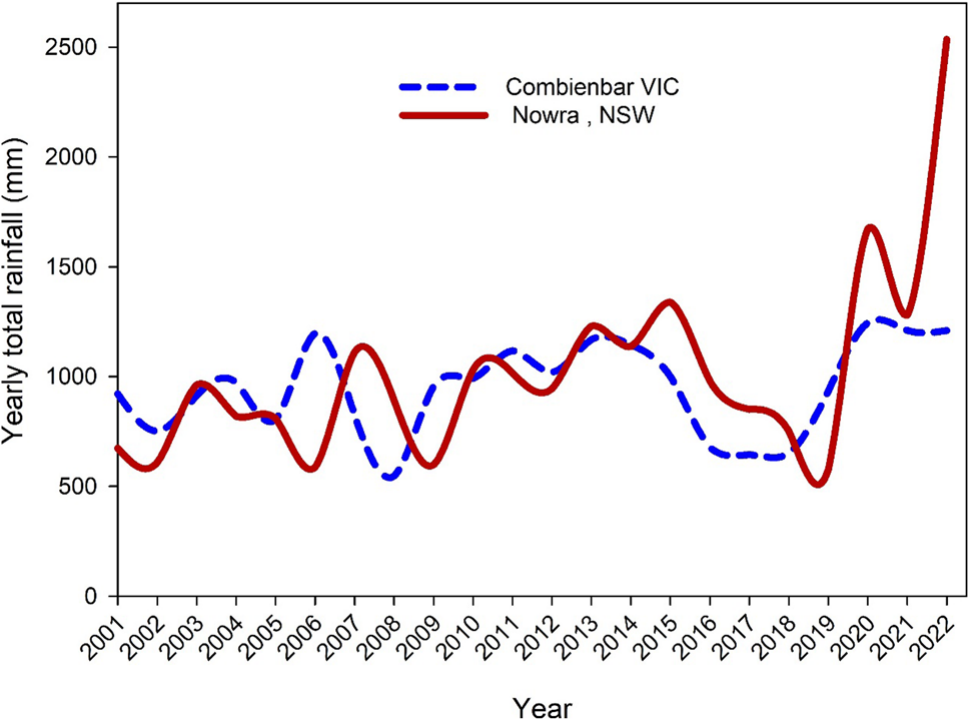
Total rainfall (mm) during 2001–2022 at Combienbar VIC and Nowra NSW. Data obtained from Australian Bureau of Meteorology

Along with temperature escalation, rainfall pattern was also an influencing factor of recent mega-fires. The rainfall data of Australia was collected from the Special Statement published by the Bureau of Meteorology for the 1999–2020 time span. Rainfall data has been represented in this study every 2 years. Rainfall was 578.8 mm at the start of the time period, and it continued to climb steadily until it reached its all-time high of 710.6 mm in the year 2000. The rainfall pattern showed a sharp decrease after 2000 and a fluctuating trend until 2009. Then, again, there was a rising trend reaching 683.7 mm in 2010 and 696.7 mm in 2011 (ACS 2020). The lowest rainfall in Australian history was observed in 2019 (Fig. 4). Such a dry season with minimum rainfall ignited 2019/2020 bushfires in Australia as a disaster (Filkov et al. 2020a, b). The 2019–2020 bushfire seasons began with a lack of rainfall in large swaths of eastern Australia. The unexpectedly low rainfall in 2019 resulted in significant moisture shortages year-round (Bureau of Meteorology (BoM) 2019). The low moisture content is experienced in the Murray–Darling Basin (Filkov et al. 2020a, b). The average annual soil moisture record in five of the Basin’s 26 river catchments was the lowest for the year 2019, and after 2018 and 2002, it was the third-lowest on-record value for the Basin as a whole. The year 2019 was also the driest year on record for the Basin (ACS 2020). The below-average precipitation that fell throughout the reserving season also had an effect on coastal New South Wales, eastern South Australia, eastern Victoria, northwestern Victoria, the east coast and north coast of Tasmania and the south west region of Western Australia. Rainfalls in New South Wales, Victoria and South Australia were the lowest in their history in the 2019/2020 season, while Western Australia and Northern Territory faced 2nd most poor rainfall records in history (ACS 2020; Filkov et al. 2020a, b), eventually bringing a suitable environment for this horror bushfire, ‘Black Summer’.

**Fig. 5:**
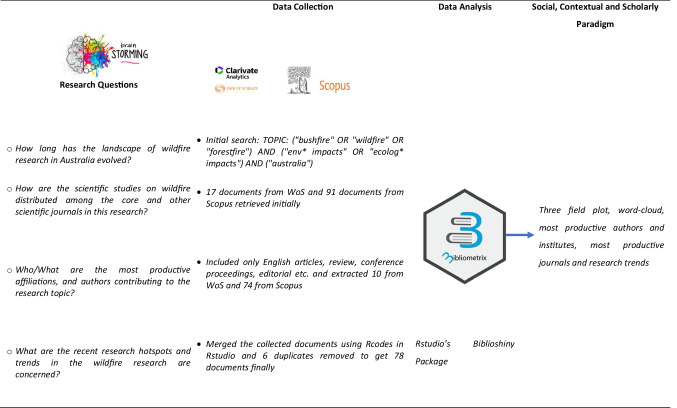
Conceptual design of the current bibliographic study

Wildfire causes damage to almost every environmental component in a way that is irreplaceable to some extent. Considering the aforementioned wildfire incidence and incurred damages, we aimed to perform a bibliometric analysis for Australia. Bibliometric analysis is the most typical non-traditional review tool. It is a collection of mathematical and statistical techniques for displaying current and ongoing knowledge on a study topic. This tool allows for the collection of reliable quality indicators. It can detect research trends based on country/region publishing outputs, author profiles and research institutes to create an overall research perspective on a subject of interest. The distribution of words in the article’s headline and the keywords can also be used to compare research patterns across time. The current study’s conceptual design is depicted in Fig. 5.

## Methodology

We gathered all of the available data about the number of fires, areas under fire disaster, lives lost and homes lost from a large compilation of news stories, survey reports, media releases from responsible authorities of the Australian Government and a few published papers taken from two reliable scientific databases: Scopus and Web of Science. Our goal was to understand the severity of Australian bushfires over the past two decades. In addition, this research aimed to understand the impacts of several fires on burned areas and homes loss to lives lost in the most affected New South Wales and Victoria states during ‘Black Summer’ and to assess the strength and direction of the relationship between the number of the fire, fired up area, homes and lives losses.

### Data sources for bibliometric analysis

Several databases offer indexed journal articles, including Google Scholar, Scopus, Web of Science (WoS) and others. Google Scholar has been criticised for admitting works from predatory journals that do not validate their originality or follow basic editorial norms (Ibba et al. 2017; Chapman and Ellinger 2019). Moreover, due to its lack of quality assurance and irregular citation counts, Google Scholar is unsuitable as a bibliometric tool (Aguillo 2012). WoS was the first collection to offer and permit bibliometrics study, covering 1900 to the present (Mingers and Leydesdorff 2015). Compared to Google Scholar and Scopus, WoS asserts that their collection is the most comprehensive and includes papers with high impact factors (Aghaei Chadegani et al. 2013). The WoS database is distinctive, contains all sorts of articles and recognises their contributors and bibliographic citations (Mongeon and Paul-Hus 2016).

Conversely, Scopus is the world’s most comprehensive reference and abstract repository for the peer-reviewed study of science, engineering, pharmacy and sociology. Elsevier, Springer, Emerald, Interscience and Taylor & Francis are among the publishers having over 20,000 peer-reviewed journals (Fahimnia et al. 2015). Scopus is a well-known scholarly repository for literature and research findings, with both WoS and Scopus-indexed academic publications (Falagas et al. 2008; Oakleaf 2009). We decided to explore key terms and keywords in Scopus and WoS repositories in this study, based on the recommendations of Fernández et al. (2010) and Mongeon and Paul-Hus (2016) by combining Scopus and WoS records.

## Topic search, data capture and mining methods

WoS and Scopus were utilised between 1 and 15 January 2022, to locate all essential papers regarding bushfires/wildfires published between 1999 and 2021. Before 1999, our preferred databases found no published works on wildfires. Because 2022 was not available at the time of the study, articles listed in both repositories following 31 December 2021, were excluded. The following search string was used to conduct the queries: TOPIC keywords: (‘bushfire’ OR ‘wildfire’ OR ‘forestfire’) AND (‘env* impacts’ OR ‘ecolog* impacts’ OR ‘human health* impacts’) AND (‘australia’). A topic keyword search includes the title of the article, buzzwords and summary. It was important to verify that the search would be conducted using the correct search word; therefore, we utilised quotation marks. Boolean operators were used in order to guarantee that each and every document was gathered. The Boolean operators used were ‘OR’ and ‘AND’, with the former ensuring that any relevant keywords are detected. The terms in the first set of brackets, however, are only pertinent to the terms in the text.

Both databases have been updated to incorporate citations for English-language research publications, literature reviews and conference/paper proceedings. After that, researchers manually eliminated documents that did not fit our criteria and those that did not have the authors’ names, abstracts or complete text. The revised papers were collected from WoS and Scopus as.txt and.bib files, respectively. Both files were combined using Rcodes in Rstudio, and six duplicate records were detected and eliminated. Finally, 78 records were gathered for the bibliometric analysis (see Table 7 for the most prominent research/review articles on Australian wildfire and its impact).

### Data analysis

Bibliometric analysis can be performed using various advanced tools and software. The most commonly used software includes Gephi, BibExcel, VOSviewer, Histcite, Pajek, Citespace and Biblioshiny (the bibliometrix package in Rstudio). For Scopus data, Histcite does not provide bibliometric analysis (Fahimnia et al. 2015). BibExcel operates in a complicated environment requiring knowledge and expertise to do a simple analysis (Fahimnia et al. 2015). We also discovered that accurately using the merged data in Citespace was impossible. For these reasons, we used the well-known statistical computing software R (Biblioshiny in this case) to do the bibliometric analysis in this work. R is open-source and free software that includes several packages for bibliometric analysis (Firdaus et al. 2019).

The bibliometrix tool in R, Biblioshiny, is particularly user-friendly for those unfamiliar with coding (Aria and Cuccurullo 2017). The program yielded data on the most productive authors, countries/regions, institutions, conceptual structure, research hotspots, social structure, and intellectual structure in wildfire research. In addition, the authors’ co-citation network was extracted as a Pajek file from Biblioshiny and displayed with VOSviewer for enhanced visualization.

### Measure of influence

In 2005, Hirsch devised objective criteria for evaluating a person’s scientific productivity (Hirsch 2005). An individual is associated with publications in this context, including an author, country/region, institution, journal and so on. The h-index measures how many times h of a person’s publications have been cited at least h times over a given period (Braun et al. 2006). For example, an author’s h-index is 20 if he or she has 20 articles with at least 20 citations. This metric was used in addition to the usual cumulative number of citations and published articles in the current study. Eugene Garfield invented the impact factor (IF) in 1972 as a complement to the h-index. This is a special kind of efficiency measure that appears only in scholarly publications. It is a measure used by journals that shows how often their articles are cited on average over 2 years. Since the impact factor is strongly correlated with the calibre of the research published in a certain journal, it is often used as a measure of both the quality of the research and the relevance of the study itself (Mao et al. 2015).

## Results and discussion

### Summary information

The dataset that was studied in the literature is summarised along with some basic statistics. In order to provide a comprehensive overview of wildfires in Australian literature, it is required to provide such a picture. Table 4 summarises the key findings from 78 publications between 1999 and 2021. The literature entries in the dataset come from 49 distinct sources, including various journals, conference papers, editorials, letters, reviews and brief surveys, to name a few. There are 297 authors in the dataset, 10 of whom single-authored 12 pieces of literature and 287 among whom co-authored articles with others. The document indicates that, on average, there were 3.81 writers and 4.22 co-authors. The dataset contains a total of 262 identified author keywords and 970 Keywords Plus entries. The latter part of this article delves more into the academic development of the research area over the course of the last two decades.

**Table 4:** Main information about the final and merged dataset

Description	Results
Main information about data	
Timespan	1990:2021
Sources (journals, books, etc.)	49
Documents	78
Average years from publication	6.83
Average citations per documents	23.83
Average citations per year per doc	3.461
References	4404
Document types	
Article	63
Conference paper	3
Editorial	3
Letter	1
Note	2
Review	4
Short survey	2
Document contents	
Keywords plus (ID)	970
Author’s keywords (DE)	262
Authors	
Authors	297
Author appearances	329
Authors of single-authored documents	10
Authors of multi-authored documents	287
Authors’ collaboration	
Single-authored documents	12
Documents per author	0.263
Authors per document	3.81
Co-Authors per documents	4.22
Collaboration index	4.35

### RQ1: How long has the landscape of wildfire research in australia evolved?

**Fig. 6:**
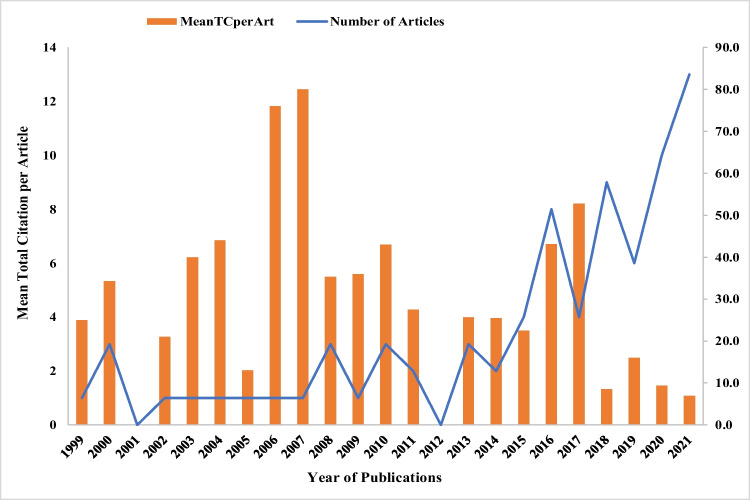
Evolution of wildfire research from 1999 to 2021

Despite occasional fluctuations over the study timeframe, the cumulative number of publications climbed steadily, as seen in Fig. 6. There was a steady state from 2002 to 2007 with only one publication each year. There were no publications in 2001 and 2012, and the highest number of articles was published in 2021 (13 articles). It is conspicuous that since 2015, the interest in wildfire-related research has increased. The research field is rapidly expanding by 13.68% every year. So far, three distinct evolutionary phases have been identified: early evolution (8 articles from 1999 to 2005), sluggish evolution (from 2006 to 2013, there were 14 publications) and rapid evolution (56 publications from 2014 to 2021). When comparing articles from the beginning of evolution to those from the slow and rapid evolution periods, the cumulative growth rates are 75% and 600%, respectively. It is unsurprising that after 2015, the number of articles has increased dramatically, as some major wildfires occurred and 2019/2020 ‘Black Summer’ megafires got considerable attention among researchers. The mean total citations per article (MeanTCperArt) pinnacled in 2007 (80), followed by 76 in 2006, and no citations were counted in 2001 and 2012. Since 2018, there has been a considerable reduction in citations, owing to the fact that it takes many years for recently published works to obtain significant citations. This section’s trend shows how communication and investigation in this area of science are inciting the scientific community’s interest. This is a positive step toward wildfire research and fire management.

### RQ2: How are the scientific studies on wildfire distributed among the core and other scientific journals in this research?

The source of the articles was investigated in order to establish which journals had the greatest number of publishing. The top ten highest prolific journals are listed in Table 5. The journals Forest Ecology and Management and Science have the most publications, accounting for 7.70% of all wildfire-related papers from 1999 to 2021. For journals that publish articles on the study’s topic, it is useful to look at the number of publications as well as other indices like impact factor, total citations (TC), and h-index. Despite having low number of publications (NP = 2) in Climatic Change, in terms of TC, it has garnered considerable attention (116) after Forest Ecology and Management (NP = 6, TC = 214) and PLOS ONE (NP = 5, TC = 143). This could be attributed to the fact that Forest Ecology and Management is the journal with the earliest publication year (PY = 2004), while Climatic Change started its publications in 2016 and PLOS ONE in 2011. The correlation among NP, h-index and TC is pretty significant and positive.

**Table 5:** Performance of top 10 most productive journals

Element	IF	h-index	TC	NP	PY-start
Forest Ecology and Management	3.558	4	214	6	2004
Science	41.84	5	60	6	2018
PLOS ONE	3.24	4	143	5	2011
Science of the Total Environment	7.963	4	96	4	2016
Journal of Environmental Management	6.789	3	82	3	2018
The Lancet Planetray Health	19.173	1	8	1	2020
Australian Forestry	1.9	2	38	2	1999
Climatic Change	4.743	2	116	2	2016
Fire	N/A	1	9	2	2021
International Journal of Wildland Fire	2.627	2	17	2	2018

*NP* number of publications, *TC* total citations, *IF* impact factor, *PY* publication year, *N/A* not available

**Fig. 7:**
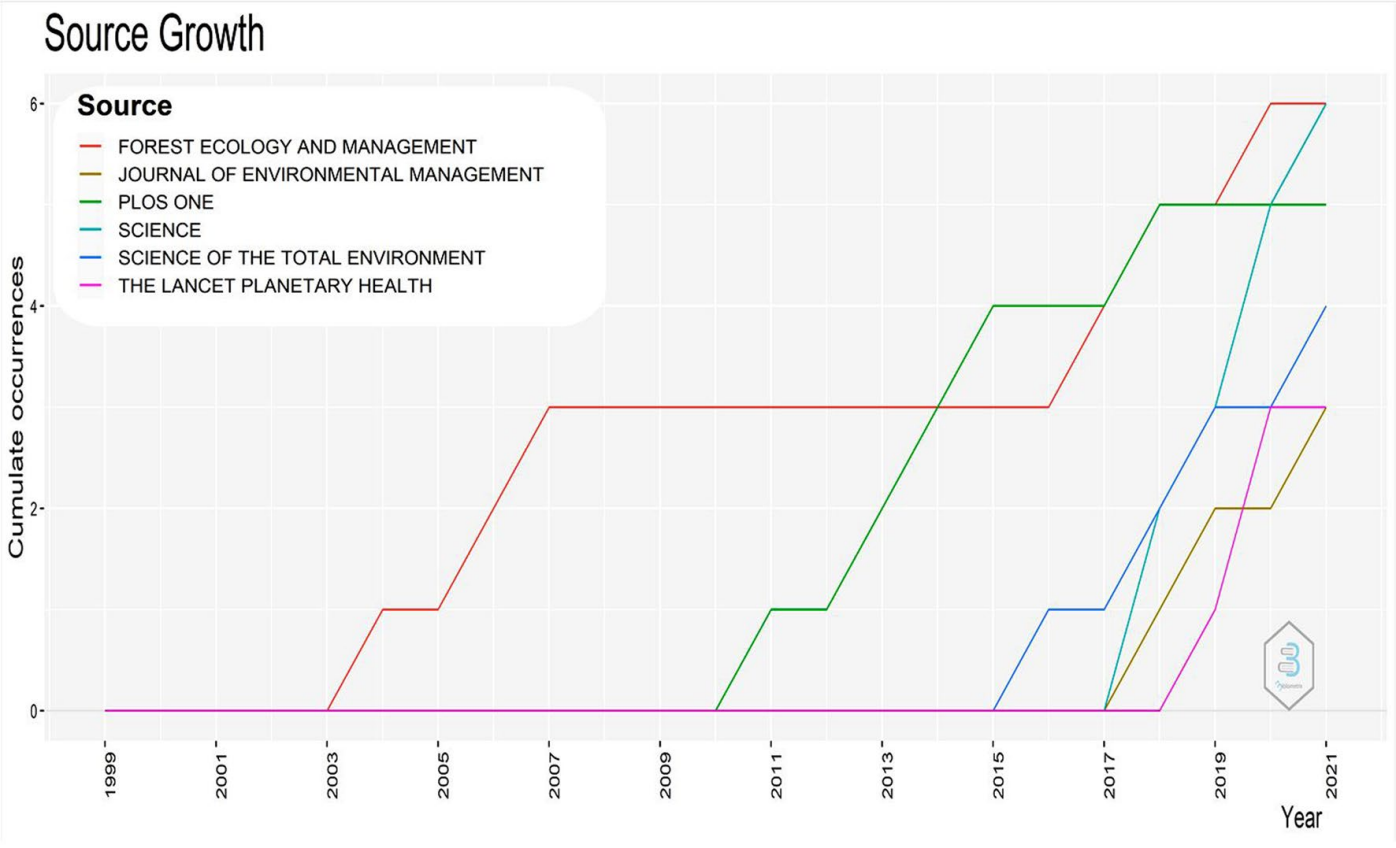
Distribution of publications on wildfires across the top six journals

Regarding the impact factor (IF), Science (41.84), The Lancet Planetary Health (19.173), Science of the Total Environment (7.963) and Journal of Environmental Management (6.789) established as the publications that include high-quality scientific writings that have been peer-reviewed. The top six journals’ progression throughout time is depicted in Fig. 7. There were little scholarly efforts on the issue in these journals from 2000 to 2004. Forest Ecology and Management was the most widely published journal on this subject from 2004 to 2014, when PLOS ONE temporarily surpassed it. However, Forest Ecology and Management reclaimed its status as the premier publication in this field from 2020 to 2021. In the 2020–2021 period, Forest Ecology and Management, Science, PLOS ONE and Science of the Total Environment were the leading journals regarding productivity.

### RQ3: What organizations and people have done the most to advance knowledge in this field?

**Fig. 8:**
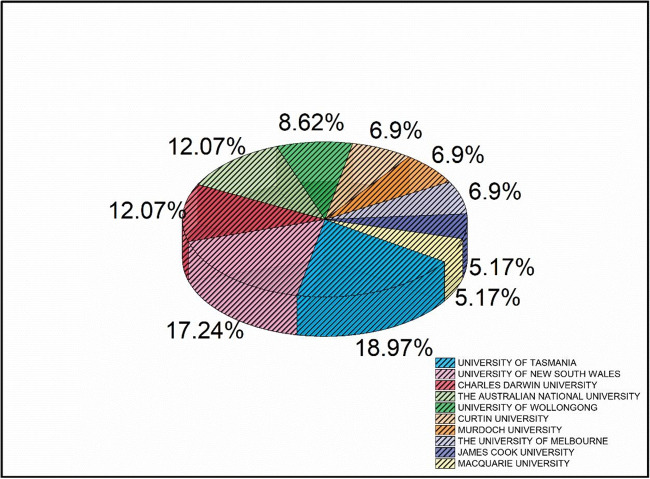
Top 10 Australian institutions according to number of published articles

Furthermore, from 1999 to 2021, on the topic of wildfire research, a total of 124 research institutions contributed. About 8.06% of all organizations have published at least three publications. This shows that only a few Australian organizations are actively driving this field of research. Figure 8 depicts the outputs of the top 10 organizations in this study that display the contributions of the most relevant institutions in wildfire research in Australia. With 18.97% of the publications produced by these ten institutions, the University of Tasmania has been the most prolific, followed by the University of New South Wales (17.24%). The third most productive institutions were found Charles Darwin University (12.07%) and The Australian National University (12.07%) with a similar number of articles. According to the information gathered, 297 authors have written at least one article about wildfires between 1999 and 2021. Of these authors, 7.41% have at least two publications, and others produced single publications. Table 6 lists the top ten authors on the subject of wildfire research. These ten authors have combined produced 54 of the 329 documents retrieved (16.41%). It is seen that D. Lindenmayer from The Australian National University (ANU) is the writer with the largest publication and multiple performance indicators are implemented in the field; it is found to be the most productive. He has the maximum overall citations as well as the highest h-index. As a result, we endeavoured to figure out what about this author made them so successful in their area. The author has four publications, the highest in wildfire research from 2011 to 2019. In addition, he is working as an Ecology and Conservation Biology professor at ANU, having long experience in his sector. These factors may account for the author’s greater interest and dominance in the field.

**Table 6:** Authors’ productivity

Author	Affiliation	h-index	TC	NP	PY-start
Lindenmayer, D	The Australian National University	4	167	4	2011
Banks, S	Charles Darwin University	3	138	3	2011
Blair, D	The Australian National University	3	126	3	2011
Burrows, N	Department of Conservation & Land Management	2	24	3	2000
Dixon, K	Curtin University	3	129	3	2006
Mcburney, L	The Australian National University	3	126	3	2011
Penman, T	University of Melbourne	2	130	3	2007
Sharples, J	University of New South Wales	2	102	3	2015
Binns, D	University of New South Wales	2	129	2	2007
Evans, J	Charles Darwin University	2	116	2	2016

**Fig. 9:**
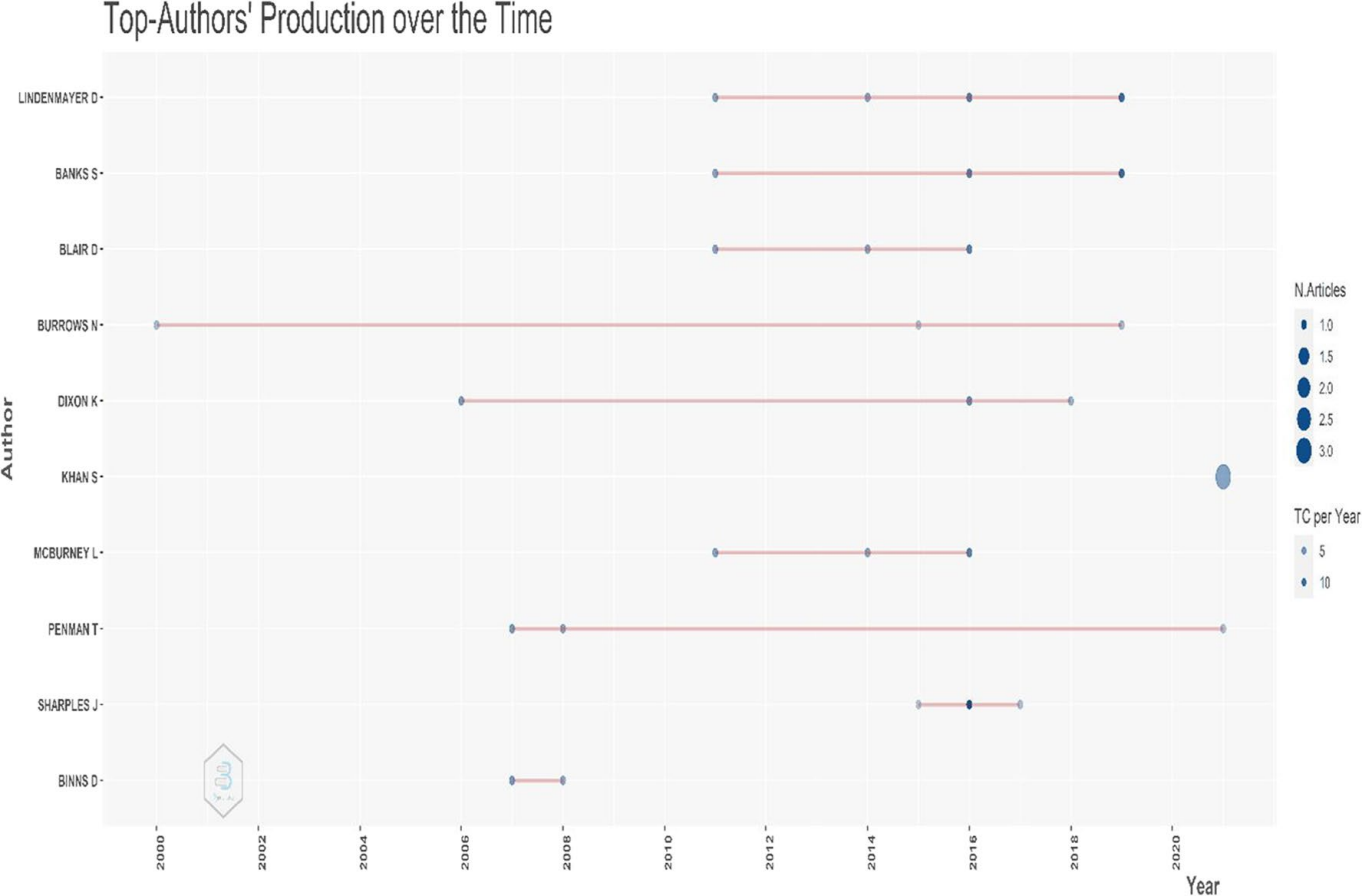
Authors’ scientific production over time

**Fig. 10:**
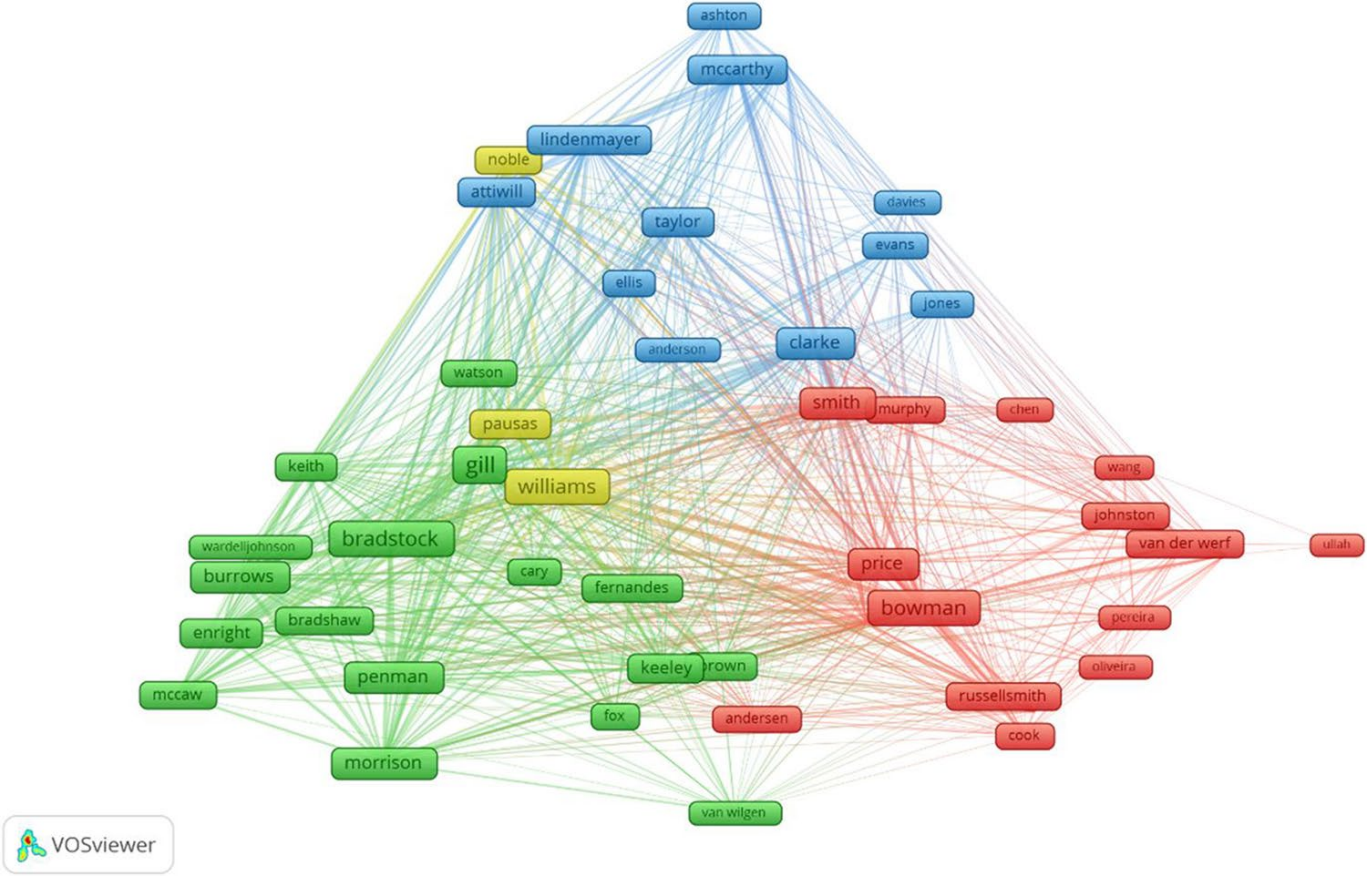
Authors’ collaboration network analysis

**Fig. 11:**
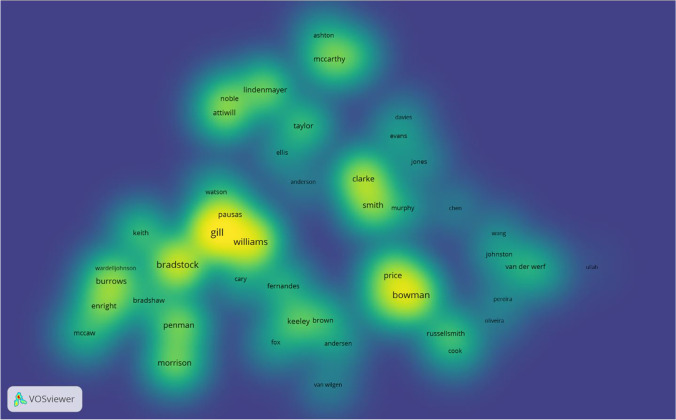
Density visualization of authors collaboration network

Figure 9 shows the annual scientific output of the ten most important researchers. The larger circles imply that there were more publications during that time. The darker the hue of the circles, the more citations of the published articles there are. The first and most recent publications of the most productive writer, D. Lindenmayer, were published in 2011 and 2019, respectively, as shown in Fig. 9. The authors with the most significant contributions in this discipline are N. Burrows and T. Penman. It is worth noting that only N. Burrows was among the first to contribute to the field (from 2000). Figure 10 shows the research collaboration of authors from the same/different institutions by looking at the linkages among the co-authors listed in the publications. There are four clusters found consisting of 49 authors. The largest cluster (green) consists of 17 authors, the red cluster has 17 authors, 12 from blue and the fewest (three) in yellow clusters. Figure 11 shows the density visualization based on the author’s collaboration network with the colour spectrum. It shows that two clusters are highly dense, composed of authors ‘Price’, ‘Bowman’ and ‘Bond’ and another is ‘Pausas’, ‘Gill’ and ‘Williams’. There are also six light yellow-coloured dense clusters as seen in Fig. 11 centred by ‘Clarke’, ‘Burrows’, ‘Penman’, ‘Noble’, ‘Lindenmayer’ and ‘Mccarthy’. Authors who have more collaborations with others are visualised as red marked clusters followed by green and blue. It can also be seen with a more dense colour spectrum in density visualization analysis for more productive authors with higher collaboration. To increase the research outcomes on the topic of wildfire research in Australia, researchers should be encouraged to join international and national collaborations.

### RQ4: Where is the current wildfire research in australia focusing, and what are the emerging trends?

Keywords Plus is used in this part to find research hotspots and trends in wildfire studies. Words or phrases that often occur in the titles of citations inside an article but not in the titles themselves or as Author Keywords are considered Keywords Plus. Garfield (1990) claimed that Keywords Plus terms might represent the contents of the article at a deeper level and with more diversity, whereas Zhang et al. (2016a, b) suggested that Keywords Plus should be used in scientific disciplines’ bibliometric analysis.

**Fig. 12:**
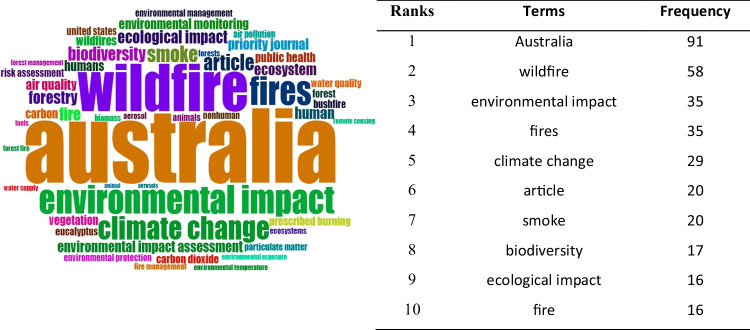
Top 50 keywords on wildfire research from 1999 to 2021

**Fig. 13:**
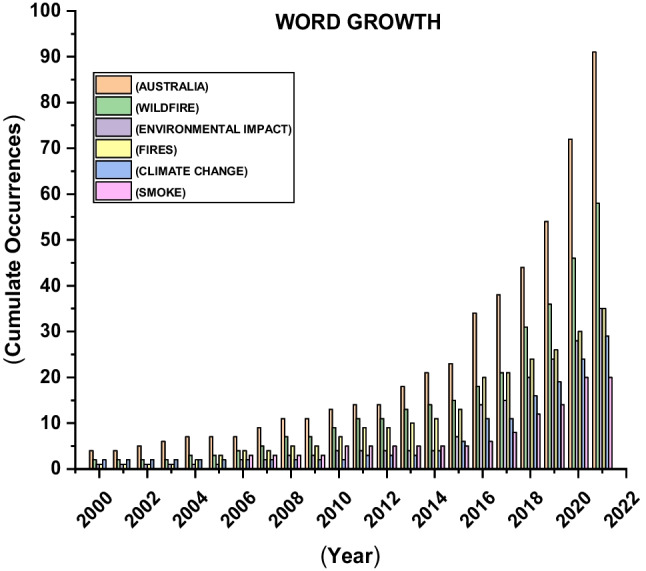
Top six Keyword Plus growth from 2000 to 2021

Figure 12 highlights the fifty most regularly mentioned phrases in the research field. The most often occurring phrases are ‘Australia’ and ‘wildfire’, followed by ‘environmental impact’ and ‘fires’. The frequency of the top 10 Keywords Plus is found at least 16 times, and the observation suggests that all of them have mainly centred on the fire, smoke, and environmental impacts of wildfire. Unsurprisingly, the term ‘Australia’ got the highest frequency (91), and ‘wildfire’ got the second highest (58) because most research articles focused on fire or bushfires. The authors are assessing ecological or environmental impacts after wildfires in Australia. This trend is presently escalating with the increasing interest of researchers. Figure 13 shows the top six Keyword Plus growth from 2000 to 2021. ‘Australia’, ‘wildfire’, ‘environmental impact’, ‘fires’, ‘climate change’ and ‘smoke’ are mostly abundant and have the highest growth in between the study timespan. ‘Australia’ is the most frequent and has maximum growth followed by ‘wildfire’ and ‘fires’. From 2000 to 2015, the growth was slow, but from 2016, the word growth escalated significantly till 2019, and after that period, research intensity was maximum and got the highest frequency. It shows that research interest in wildfires or fires has increased dramatically in recent times in Australia.

## Wildfire management in Australia

Wildfires, including droughts, have long been a part of the Australian climate (Bell and Adams 2008; Borchers Arriagada et al. 2020; Dickman 2021), which are now a significant environmental and socioeconomic threat, with government agencies in Australia and New Zealand spending hundreds of millions of dollars per year to combat them (Filkov et al. 2020a, b). The number of people living on the urban–rural interface in bushfire-prone areas is rising significantly each year, thanks to expanding capital and regional cities and better lifestyle. Wildfire-related disasters have made them victims (Eriksen and Gill 2010; Fairbrother et al. 2013). The raw materials for any wildfire are the availability of fuel such as grass, leaves and twigs of plants, oxygen from the ambient air and heat or direct flame (Chuvieco et al. 2002; Aldeias et al. 2016). However, Australia should reduce the likelihood of a fire and limit the spread by reducing the raw materials responsible for wildfires. Land management techniques could be one of Australia’s significant options for fire risk management (Hughes and Mercer 2009; Syphard et al. 2013). Reducing forest or grassland fuel presence (Price et al. 2015), slower and often stopped bushfires spread (Ellis et al. 2004) and offering firefighters better access routes to reach the blazing locations easily might be effective during the fire (Dwyer 2022). After a fire, land management is a crucial factor to minimise the losses and intensity of fires (Garcia et al. 2021). A community-based approach is also needed for land management strategies for firefighting across Australia (Russell-Smith et al. 2017). Rural people or people who live near the bushland in urban areas have both their own, their neighbours and the broader community are the key stakeholders to land management and fire prevention (Hughes and Mercer 2009; Koksal et al. 2019).

To make buildings or houses more resistant to fire hazards, strong building codes and regulations should be established for each Australian state (Hamin and Gurran 2009; Mutch et al. 2011; Navaratnam et al. 2019). Local government authorities in some fire-prone states have rules governing home siting, layout, and the use of construction materials (Hughes and Mercer 2009; Mockrin et al. 2020). The authority should keep an eye on implementing all building design and planning requirements. These measures can be effective in minimizing damage to houses and reducing fire losses and preventing and spreading bushfires (Gill et al. 2013; Calkin et al. 2014). Most bushfires in Australia are caused by people and their acts as intentional or unintentional burning-off that has gotten out of hand as well as fires escaping from burning garbage heaps (Thakur 2023). Mass education is usually intended to give people a greater understanding of the consequences they face from wildfires and the steps the community may take to reduce the risks. Television and radio programs could be useful in informing the general public about their duties in terms of fire prevention (Folkman 1973; McCaffrey 2004). These fire safety and prevention campaigns could be aired throughout the year, especially for fire-prone states across Australia.

Moreover, they can inform citizens about the impending danger to adopt necessary measures. Besides, responsible authorities of different states should focus on the following measures and strongly implement the guidelines. First, the public entrance should be restricted to the forest land (Black et al. 2013). Better soil management should be considered to keep the ecosystem alive; for example, increasing soil microbial activities to reduce soil erosion (Lal 2015). Third, an eco-friendly fuel policy should be accepted to reduce the temperature (Haque et al. 2021). Creating wildlife sanctuaries to protect endangered species in their natural habitat should be essential for reducing risk management. Environmentally sound development projects should be planned to minimise fire risk (Distefano 2005). However, there are several challenges for researchers and policymakers to understand the magnitude of fire threats and design practical management approaches (Stephens and Ruth 2005; Cosgrove and Loucks 2015). There are lack of data and trustworthy research articles, insufficient fire impact assessment study and inadequate study on cause-effect relationship of fires; these are some challenges for researchers to forecast before fire seasons (Paoletti et al. 2007). Bushfires also have a number of additional management challenges. The challenge is to provide stakeholders with reliable information on rate of fire spread and location of the fire front so that they can plan secure preparation time in their specific circumstances (Gill and Stephens 2009). Another challenge is predicting the effects of fires on various flora and fauna species composition (Chapin 2003; Gill et al. 2013). These challenges should be addressed by responsible authorities in Australia with the goal of ‘long-term improvements rather than short-term fixes of the system’.

## Research gaps

There are a few gaps in the field’s existing literature, according to the titles and abstracts of the 78 publications recovered and the conclusions of the current study (Table 7). The first research gap is the scarcity of studies on management policies and regulatory systems to limit wildfires’ size. Most of the published articles were based on wildfire causes and consequences. Second, most research focuses solely on fires’ ecological and environmental effects but does not significantly discuss human health impacts. According to (Wintle et al. 2020), the mega-fires in Australia during 2019–2020 resulted in the devastating loss of human life, the worst destruction of habitats for endangered species and damage to ecological communities in postcolonial history. They studied to protect impacted species from extinction and showed how to avoid repeating the impacts of such devastating bushfires. A holistic bushfire evaluation and mitigation model have been suggested based on a mixed-method approach of Geographical Information Systems (GIS), remote sensing, and unmanned aerial vehicles (UAV) (Munawar et al. 2021a, b); however, this is the most recent notable investigation about fire management after the study conducted by Kanowski et al. (2005). These studies may be important for responsible authorities to adopt the proper fire impacts mitigation and management policies. Another research gap is the small amount of study done in this area. Though our study in the ‘Summary and information’ section (RQ1) indicates that the research field is expanding, the annual growth rate is not encouraging. Finally, there is still potential for development in Australia regarding inter-institution/author joint research.

**Table 7:** List of publications related to Australian bushfire from 1994 to 2023

Titles	Year	Source title	Document type	References
Management of forest fire in Australia and fire ecology	1994	Chinese Journal of Applied Ecology	Article	DM and Guo (1994)
Ecological effects of firefighting foams and retardants: A summary	1999	Australian Forestry	Article	Adams and Simmons (1999)
The effect of three fire regimes on stream water quality, water yield and export coefficients in a tropical savanna (northern Australia)	2000	Journal of Hydrology	Article	Townsend and Douglas (2000)
Behaviour and some impacts of a large wildfire in the Gnangara maritime pine (Pinus pinaster) plantation, Western Australia	2000	CALM Science	Article	Burrows et al. (2000)
The biodiversity crisis and adaptation to climate change: A case study from Australia’s forests	2000	Environmental Monitoring and Assessment	Article	Williams (2000)
Impacts of logging, fire and grazing regimes on bird species assemblages of the Pilliga woodlands of New South Wales	2002	Pacific Conservation Biology	Article	Date et al. (2002)
The effects of experimental patch burning and rainfall on small mammals in the Simpson Desert, Queensland	2003	Wildlife Research	Article	Letnic (2003)
Does clearfell, burn and sow silviculture mimic the effect of wildfire? A field study and review using litter beetles	2004	Forest Ecology and Management	Article	Baker et al. (2004)
Inquiries following the 2002–2003 Australian bushfires: Common themes and future directions for Australian bushfire mitigation and management	2005	Australian Forestry	Article	Kanowski et al. (2005)
Post-fire germination: The effect of smoke on seeds of selected species from the central Mediterranean basin	2006	Forest Ecology and Management	Article	Crosti et al. (2006)
Salvage logging in the montane ash eucalypt forests of the Central Highlands of Victoria and its potential impacts on biodiversity	2006	Conservation Biology	Review	Lindenmayer and Ough (2006)
Patchiness of prescribed burns in dry sclerophyll eucalypt forests in South-eastern Australia	2007	Forest Ecology and Management	Article	Penman et al. (2007)
Changes in understorey plant species richness following logging and prescribed burning in shrubby dry sclerophyll forests of south-eastern Australia	2008	Austral Ecology	Article	Penman et al. (2008)
Mapping burned areas and burn severity patterns in SW Australian eucalypt forest using remotely-sensed changes in leaf area index	2008	Remote Sensing of Environment	Article	Boer et al. (2008)
Numerical modelling of the impact of wildland-urban interface fires on Coimbra air quality	2008	WIT Transactions on Ecology and the Environment	Article	Miranda et al. (2008)
Eucalypt smoke and wildfires: Temperature-dependent emissions of biogenic volatile organic compounds	2009	International Journal of Mass Spectrometry	Article	Maleknia et al. (2009)
Using silicone passive samplers to detect polycyclic aromatic hydrocarbons from wildfires in streams and potential acute effects for invertebrate communities	2010	Water Research	Article	Schäfer et al. (2010)
MISR stereo heights of grassland fire smoke plumes in Australia	2009	IEEE Transactions on Geoscience and Remote Sensing	Article	Mims et al. (2009)
Resourcing challenges for post-disaster housing reconstruction: A comparative analysis	2010	Building Research and Information	Article	Chang et al. (2010)
The effects of wildfire on mortality and resources for an arboreal marsupial: Resilience to fire events but susceptibility to fire regime change	2011	PLOS ONE	Article	Banks et al. (2011)
Estimating catchment-scale impacts of wildfire on sediment and nutrient loads using the E2 catchment modelling framework	2011	Environmental Modelling and Software	Article	Feikema et al. (2011)
Landscape Scale Influences of Forest Area and Housing Density on House Loss in the 2009 Victorian Bushfires	2013	PLOS ONE	Article	Price and Bradstock (2013)
Prescribed fire in eucalypt woodlands: Immediate effects on a microbat community of northern Australia	2013	Wildlife Research	Article	Inkster-Draper et al. (2013)
Estimating the economic, social and environmental impacts of wildfires in Australia Catherine Stephenson1	2013	Environmental Hazards	Article	Stephenson et al. (2013)
Accounting for biomass carbon stock change due to wildfire in temperate forest landscapes in Australia	2014	PLOS ONE	Article	Keith et al. (2014)
Climate change and the management of fire-prone vegetation in southwest and southeast Australia	2014	Geographical Research	Article	Enright and Fontaine (2014)
Deriving multiple benefits from carbon market-based savanna fire management: An Australian example	2015	PLOS ONE	Article	Russell-Smith et al. (2015)
Trade-offs in adaptation planning: Protecting public interest environmental values	2015	Journal of Environmental Law	Article	Foerster et al. (2015)
A rate of spread index for fires in spinifex fuels	2015	Proceedings—21st International Congress on Modelling and Simulation, MODSIM 2015	Conference paper	Sharples et al. (2015)
Ecological effects of extreme climatic events on riverine ecosystems: Insights from Australia	2015	Freshwater Biology	Article	Leigh et al. (2015)
Breakdowns in coordinated decision making at and above the incident management team level: An analysis of three large-scale Australian wildfires	2015	Applied Ergonomics	Article	Bearman et al. (2015)
A long-term experimental case study of the ecological effectiveness and cost-effectiveness of Invasive plant management in achieving conservation goals: Bitou bush control in Booderee National Park in Eastern Australia	2015	PLOS ONE	Article	Lindenmayer et al. (2015)
Characterisation of the impact of open biomass burning on urban air quality in Brisbane, Australia	2016	Environment International	Article	He et al. (2016)
Disturbance gradient shows logging affects plant functional groups more than fire	2016	Ecological Applications	Article	Blair et al. (2016)
An investigation of future fuel load and fire weather in Australia	2016	Climatic Change	Article	Clarke et al. (2016)
Soil respiration dynamics in fire affected semi-arid ecosystems: Effects of vegetation type and environmental factors	2016	Science of the Total Environment	Article	Muñoz-Rojas et al. (2016)
Natural hazards in Australia: extreme bushfire	2016	Climatic Change	Article	Sharples et al. (2016)
Determining fire dates and locating ignition points with satellite data	2016	Remote Sensing	Article	Benali et al. (2016)
Spatial planning and changing landscapes: a failure of policy in peri-urban Victoria, Australia	2016	Journal of Environmental Planning and Management	Article	Llausas et al. (2016)
Effects of climate on the size of wildfires in the Eucalyptus camaldulensis forests and the dry lands of the Riverina Bioregion, Australia	2017	Forest Ecology and Management	Article	Zhang et al. (2017)
Understanding and managing the health impacts of poor air quality from landscape fires	2017	Medical Journal of Australia	Article	Johnston(2017)
Implications of floristic patterns and changes in stand structure following a large-scale, intense fire across forested ecosystems in south-Western Australia’s high-rainfall zone	2017	Pacific Conservation Biology	Article	Wardell-Johnson et al. (2017)
Human exposure and sensitivity to globally extreme wildfire events	2017	Nature Ecology and Evolution	Article	Bowman et al. (2017)
Prescribed burning reduces the abundance of den sites for a hollow-using mammal in a dry forest ecosystem	2018	Forest Ecology and Management	Article	Flanagan-Moodie et al. (2018)
Impact of intense disturbance on the structure and composition of wet-eucalypt forests: A case study from the Tasmanian 2016 wildfires	2018	PLOS ONE	Article	Lunn et al. (2018)
Prescribed fire and its impacts on ecosystem services in the UK	2018	Science of the Total Environment	Article	Harper et al. (2018)
Understanding the long-term impact of prescribed burning in mediterranean-climate biodiversity hotspots, with a focus on south-Western Australia	2018	International Journal of Wildland Fire	Review	Bradshaw et al. (2018)
Improved spatial organisation of sensor networks to reduce wildfire impact	2018	International Journal of Emergency Management	Conference paper	Budden et al. (2018)
Bushfires and indoor built environments	2018	Indoor and Built Environment	Editorial	
Perturbations have minor impacts on parasite dynamics and body condition of an endangered marsupial	2018	Journal of Zoology	Article	Jones et al. (2018)
Fine-scale temporal turnover of jarrah forest understory vegetation assemblages is independent of fire regime	2019	Fire Ecology	Article	Burrows et al. (2019)
The legacy of colonial fire management policies on traditional livelihoods and ecological sustainability in savannas: Impacts, consequences, new directions	2019	Journal of Environmental Management	Article	Moura et al. (2019)
Australian blazes will ‘reframe our understanding of bushfire’	2019	Science	Note	Pickrell(2019)
Effect of thinning and burning fuel reduction treatments on forest carbon and bushfire fuel hazard in Eucalyptus sieberi forests of South-Eastern Australia	2019	Science of the Total Environment	Article	Volkova and Weston (2019)
Long-term impacts of wildfire and logging on forest soils	2019	Nature Geoscience	Article	Bowd et al. (2019)
Record breakers	2019	The Lancet Planetary Health	Editorial	Health (2019)
The 2019 report of the MJA–Lancet Countdown on health and climate change: a turbulent year with mixed progress	2019	Medical Journal of Australia	Article	Beggs et al. (2019)
Bushfires in Australia. A review of the extreme fire season 2019/2020; [Buschbrände in Australien: Ein Rückblick auf die extreme Feuersaison 2019/2020]	2020	Geographische Rundschau	Article	Sebastian Fastenrath (2020)
Atmospheric remobilization of natural and anthropogenic contaminants during wildfires	2020	Environmental Pollution	Article	Isley and Taylor (2020)
Record U.S. And Australian fires raise fears for many species	2020	Science	Note	Pickrell and Pennisi (2020)
Fire and rust–the impact of Austropuccinia psidii (myrtle rust) on regeneration of Myrtaceae in coastal heath following wildfire	2020	Southern Forests	Article	Pegg et al. (2020)
After the Megafires: What Next for Australian Wildlife?	2020	Trends in Ecology and Evolution	Short survey	Wintle et al. (2020)
Wildfire smoke, a potential infectious agent	2020	Science	Review	Kobziar and Thompson (2020)
The effects of inter-fire interval on flora-fauna interactions in a sub-alpine landscape	2020	Forest Ecology and Management	Article	O’Loughlin et al. (2020)
Assessment of the characteristics of recent major wildfires in the USA, Australia and Brazil in 2018–2019 using multi-source satellite products	2020	Remote Sensing	Article	Kganyago and Shikwambana (2020)
Long-term trends in PM2.5 mass and particle number concentrations in urban air: The impacts of mitigation measures and extreme events due to changing climates	2020	Environmental Pollution	Article	de Jesus et al. (2020)
Australia burning	2020	The Lancet Planetary Health	Article	Hope (2020)
Widespread phytoplankton blooms triggered by 2019–2020 Australian wildfires	2021	Nature	Article	Tang et al. (2021)
Mechanical mastication reduces fuel structure and modelled fire behaviour in Australian shrub encroached ecosystems	2021	Forests	Article	Grant et al. (2021)
When natural hazards intersect with public health: A preliminary exploration of the impact of bushfires and the covid-19 pandemic on Australian coastal drowning fatalities	2021	International Journal of Environmental Research and Public Health	Article	Lawes et al. (2021)
Environmental degradation & role of financialisation, economic development, industrialisation and trade liberalisation	2021	Journal of Environmental Management	Article	Nasir et al. (2021)
Ecological consequences of Australian “Black Summer” (2019–20) fires: A synthesis of Australian Commonwealth Government report findings	2021	Integrated Environmental Assessment and Management	Article	Khan (2021)
Continental risk assessment for understudied taxa post-catastrophic wildfire indicates severe impacts on the Australian bee fauna	2021	Global Change Biology	Article	Dorey et al. (2021)
UAV assisted spatiotemporal analysis and management of bushfires: A case study of the 2020 Victorian bushfires	2021	Fire	Article	Munawar et al. (2021a, b)
Beyond bushfire severity: mapping the ecological impact of bushfires on the Gondwana Rainforests of Australia World Heritage Area	2022	Australian Zoologist	Article	Laidlaw et al. (2022)
Incendiary Humor: Climate Change, Biodiversity, and Politics in Wildfire Cartoons	2022	Environmental Communication	Article	Moret-Soler et al. (2022)
A burning issue: Reviewing the socio-demographic and environmental justice aspects of the wildfire literature	2022	PLOS ONE	Review	Thomas et al. (2022)
Responding to the biodiversity impacts of a megafire: A case study from south-eastern Australia’s Black Summer	2022	Diversity and Distributions	Article	Geary et al. (2022)
Raking over the ashes: assessing the impact of fire on native fauna in the aftermath of Australia’s 2019–2020 fires	2022	Australian Zoologist	Review	Dickman et al. (2022)
Fire-related threats and transformational change in Australian ecosystems	2022	Global Ecology and Biogeography	Article	Keith et al. (2022)
How to prioritize species recovery after a megafire	2022	Conservation Biology	Article	Ward et al. (2022)
Wildfire smoke destroys stratospheric ozone	2022	Science	Article	Bernath et al. (2022)
Rapid assessment of the biodiversity impacts of the 2019–2020 Australian megafires to guide urgent management intervention and recovery and lessons for other regions	2022	Diversity and Distributions	Article	Legge et al. (2022)
The 2022 report of the MJA–Lancet Countdown on health and climate change: Australia unprepared and paying the price	2022	Medical Journal of Australia	Article	Beggs et al. (2022)
Increasing threat of wildfires: the year 2020 in perspective: A Global Ecology and Biogeography special issue	2022	Global Ecology and Biogeography	Article	Nolan et al. (2022)
Canopy cover mediates the effects of a decadal increase in time since fire on arboreal birds	2023	Biological Conservation	Article	Franklin et al. (2023)
Designing digital health applications for climate change mitigation and adaptation	2023	Medical Journal of Australia	Article	Lokmic-Tomkins et al. (2023)
The Hospitalizations for Cardiovascular and Respiratory Conditions, Emergency Department Presentations and Economic Burden of Bushfires in Australia Between 2021 and 2030: A Modelling Study	2023	Current Problems in Cardiology	Review	Ademi et al. (2022)
Long-term impacts of non-occupational wildfire exposure on human health: A systematic review	2023	Environmental Pollution	Review	Gao et al. (2023)
Drones are an effective tool to assess the impact of feral horses in an alpine riparian environment	2023	Austral Ecology	Article	Giles et al. (2023)
Vast ecosystem disturbance in a warming climate may jeopardize our climate goal of reducing CO2: a case study for megafires in the Australian ‘black summer’	2023	Science of the Total Environment	Article	Hong et al. (2023)

## Limitations of the study

It is worth mentioning that the current study is not spared from limitations. This literature review and bibliographic analysis were solely performed by focusing on wildfires in Australia and did not compare with other occurrences elsewhere. Therefore, future research opportunities exist to comprehend the situation in Australia and other nations impacted by wildfires. Furthermore, the search phrases were used at the authors’ discretion to reduce excessive contamination in the database as much as possible. However, if more relevant search phrases were included, different results might have been obtained. Nevertheless, we do not expect a considerable departure from the current study’s conclusions. By integrating numerous databases, timespan, and relevant search phrases, a future study could supplement the present study to find other minor but relevant studies.

## Conclusion

Wildfires are a common and frequent occurrence in Australia, and they have played a key role in altering the continent’s landscape for millions of years. Research related to wildfires has been growing in Australia for the last two decades. A bibliographic analysis is effective in this context to know the research status and research gaps. Bibliometric analysis successfully distinguishes and maps the accumulated scientific knowledge and subtleties of evolution in well-known domains by making sense of vast amounts of unstructured data in a systematic way. So, a well-done bibliometric study can help academics get a complete picture of the research area, find gaps in knowledge, come up with new research ideas and figure out how they want to contribute to the field, laying the groundwork for the field to move forwards in new and important ways. This study gives a list of signs that can be put together to take a useful picture for advancing wildfire research. The key data of 78 different kinds of literature published between 1999 and 2021 was obtained using bibliometric approaches from 49 sources based on the Web of Science Core Collection (SCI and SSCI) and Scopus databases. Since 2016, the research industry has grown a lot, at an average rate of 13.68% per year. This study also showed six core journals: Science, Science of the Total Environment, Journal of Environmental Management, Forest Ecology and Management, The Lancet Planetary Health and PLOS ONE on wildfires research in Australia. From 1999 to 2021, 124 research organizations contributed to wildfire studies. Only 8.06% of all institutions have produced at least three publications. From 1999 to 2021, 297 authors have published at least one paper about wildfires. Of these authors, 7.41% have at least two publications, while others have only one. To handle this topic, tremendous efforts are needed to foster more cooperation among academics from the same/different institutions. International collaboration can also aid capacity building and technology transfer for wildfire research which could be especially advantageous for countries most affected by wildfires. The current study’s findings may assist in clarifying the existing state of research and future directions for public officials and academia.

## Data Availability

All raw dataset is available to view.
